# Functional characterization of TGFβ/BMP receptors in *Echinococcus granulosus sensu stricto*: implications for parasite survival and therapeutic targeting

**DOI:** 10.1128/spectrum.01318-25

**Published:** 2025-11-26

**Authors:** Liang Li, Ning Yang, Guodong Lv, Hui Wang, Jing Li, Tuerganaili Aji, Chuanshan Zhang, Renyong Lin

**Affiliations:** 1 State Key Laboratory of Pathogenesis, Prevention, and Treatment of Central Asian High Incidence Diseases, Clinical Medical Research Institute, The First Affiliated Hospital of Xinjiang Medical University 159427, Urumqi, Xinjiang, China; 2 College of Basic Medicine, Xinjiang Medical University 659768, Urumqi, Xinjiang, China; 3 Digestive and Vascular Surgery Centre, The First Affiliated Hospital of Xinjiang Medical University 159427, Urumqi, Xinjiang, China; National Institutes of Health, Rockville, Maryland, USA

**Keywords:** *Echinococcus granulosus sensu stricto*, TGFβ/BMP receptors, interaction, LDN193189

## Abstract

Cystic echinococcosis (CE), a zoonosis caused by the larval stage of the tapeworm *Echinococcus granulosus sensu lato* (*E. granulosus s.l*.), remains a global health burden. Despite the conserved role of the transforming growth factor beta (TGFβ)/Smad signaling in cellular regulation across species, the molecular mechanisms of TGFβ/bone morphogenetic proteins (BMP) receptors in *E. granulosus sensu stricto* (*E. granulosus s.s*.) are poorly understood. The TGFβ/BMP receptors of *E. granulosus s.s*. were cloned and molecularly characterized. The positive binding interaction between receptors and their ligands was identified using the yeast two-hybrid system. To investigate the role of TGFβ/BMP signaling in *E. granulosus s.s*. development and survival, LDN193189 was employed in both the *in vitro* co-culture system and subsequent *in vivo* bioassays to investigate its pharmacological effects. Both receptors, *E. granulosus* type I receptor (EgTR1) and type II receptor (EgTR2), were characterized in *E. granulosus s.s*. and expressed during the larval stages, with *egtr2* showing peak transcriptional levels in activated protoscoleces (PSCs), 40-fold higher than those in cysts. *In situ* immunofluorescence analysis demonstrated a predominant localization of EgTR1 and EgTR2 within the tegument, suckers, and hooks of PSCs, as well as in the germinal layer of larval cysts. Yeast two-hybrid analysis revealed that EgTR2 interacted with EgTR1 and HsBMP2, but not with HsTGFβ. LDN193189 treatment significantly suppressed the viability of PSCs. *In vivo* bioassays in mice demonstrated a notable reduction in the weight of cysts in the LDN193189-treated group compared with the dimethyl sulfoxide-treated group. Our results suggest that EgTR1 and EgTR2 regulate *E. granulosus s.s*. development*,* identifying TGFβ/BMP signaling as a therapeutic target for CE.

**IMPORTANCE:**

Cystic echinococcosis (CE), a neglected zoonosis caused by *E. granulosus s.l*., inflicts severe morbidity and economic losses globally. While transforming growth factor beta/bone morphogenetic proteins (TGFβ/BMP) signaling regulates cellular processes across species, its functional role in *E. granulosus s.s.* remains unexplored. Here, we elucidate the molecular basis of TGFβ/BMP receptor signaling in *E. granulosus s.s.*, revealing that *E. granulosus* type I receptor (EgTR1) and type II receptor (EgTR2) orchestrate larval development through species-specific ligand interactions. Our findings demonstrate that EgTR2 interacts with Human BMP2 (HsBMP2), establishing a unique parasitic adaptation mechanism. Pharmacological inhibition using LDN193189 caused the suppression of parasite viability *in vitro* and reduced cyst weight *in vivo*, validating these receptors as druggable targets. This study provides the evidence linking TGFβ/BMP receptor dynamics to parasitic survival strategies, while identifying a clinically accessible therapeutic avenue for CE. The discovery bridges a critical gap in parasitic helminth biology and advances precision medicine approaches for combating this neglected tropical disease.

## INTRODUCTION

Cystic echinococcosis (CE) is caused by the larvae of the *Echinococcus granulosus sensu lato* (*E. granulosus s.l*.) (1). This zoonosis has a global distribution, with 5%–30% of the population in western China exposed to *E. granulosus s.l*. (2). CE poses a significant public health burden, with recent estimates of the global burden averaging 285,500 disability-adjusted life years (DALYs) for human CE. It is listed by the World Health Organization (WHO) as one of the 20 neglected tropical diseases, particularly impacting impoverished rural communities (1, 3). The life cycle of *E. granulosus s.l*. involves definitive hosts (dogs) and intermediate hosts (ungulates, predominantly sheep). Humans become infected through accidental ingestion of tapeworm eggs, leading to the development of fluid-filled metacestode larvae, primarily in the liver and lungs (4).

The transforming growth factor beta (TGFβ)/Smad signaling pathway is highly conserved across species, including nematodes, Drosophila, mice, and humans. This pathway regulates essential cellular processes such as proliferation, differentiation, adhesion, migration, and embryonic development (5). Components of the TGFβ/Smad pathway comprise TGFβ superfamily ligands, transmembrane serine/threonine receptors (type I/II), and intracellular Smad proteins. The TGFβ superfamily includes TGFβs, activins, inhibins, and bone morphogenetic proteins (BMPs) (6). The type I and type II transmembrane receptors share structural homology, each containing an extracellular ligand binding domain, an intracellular kinase domain and an intermediate transmembrane region. Upon activation, the TGFβ ligands bind to the type II receptor (TβRII) on the cell surface, leading to TβRI phosphorylation and subsequent intracellular signal transduction through Smad-dependent or -independent pathways, ultimately modulating transcription factor expression in the nucleus (7, 8).

The TGFβ/Smad signaling pathway has been implicated in parasite proliferation, differentiation, development, and survival. In *Schistosoma mansoni* (*S. mansoni*), host-derived TGFβ ligands activate the membrane-localized TβRII and TβRI receptors, which initiate downstream Smad signaling to regulate the expression of *S. mansoni* gynaecophoral canal protein (9). RNA interference-mediated knockdown of the activin receptor SmInAct disrupts embryogenesis of eggs, demonstrating its essential role in egg development (10). In echinococcus, five Smad homologs, including one Co-Smad (EmSmadD), two AR-Smads (EmSmadA, EmSmadC), and two BR-Smads (EmSmadB, EmSmadE), have been characterized and are phosphorylated by human TGFβ/BMP receptors (11–13). The TGFβ/BMP receptor family members of *E. multilocularis*, EmTR1-4, are activated by human TGFβ/BMP2 cytokines, leading to the phosphorylation of EmSmadB and EmSmadE (14). Furthermore, the *E. multilocularis-*derived activin-like cytokine (EmACT) drives metacestode brood capsule and protoscolex formation through the TGFβ/BMP signaling pathway (15).

Recent studies have identified components of the TGFβ/Smad pathway in *E. granulosus sensu stricto* (*E. granulosus s.s*.). Four Smad homologs, EgSmadA, EgSmadC, EgSmadD, and EgSmadE, have been identified and characterized (16–19). Among these, EgSmadE translocates to Mv1Lu cell nuclei upon treatment with hsTGFβ1 or HsBMP2 and has been shown to interact directly with EgSmadD by yeast two-hybrid and pull-down analyses (18). Despite the characterization of Smad effectors in *E. granulosus s.s*., the upstream receptors and their ligand specificity remain uncharacterized, hindering therapeutic development (20, 21).

This study characterizes EgTR1 and EgTR2 in *E. granulosus s.s*., determining their larval stages localization, protein-protein interactions, and kinase functions. We further evaluate the inhibitory and cytolytic effects of TGFβ/Smad pathway inhibitors using both *in vitro* and *in vivo* assays in *E. granulosus s.s*..

## RESULTS

### Molecular cloning and characterization reveal evolutionary conservation of TGFβ/BMP receptor homologs in *E. granulosus s.s*.

**Fig 1:**
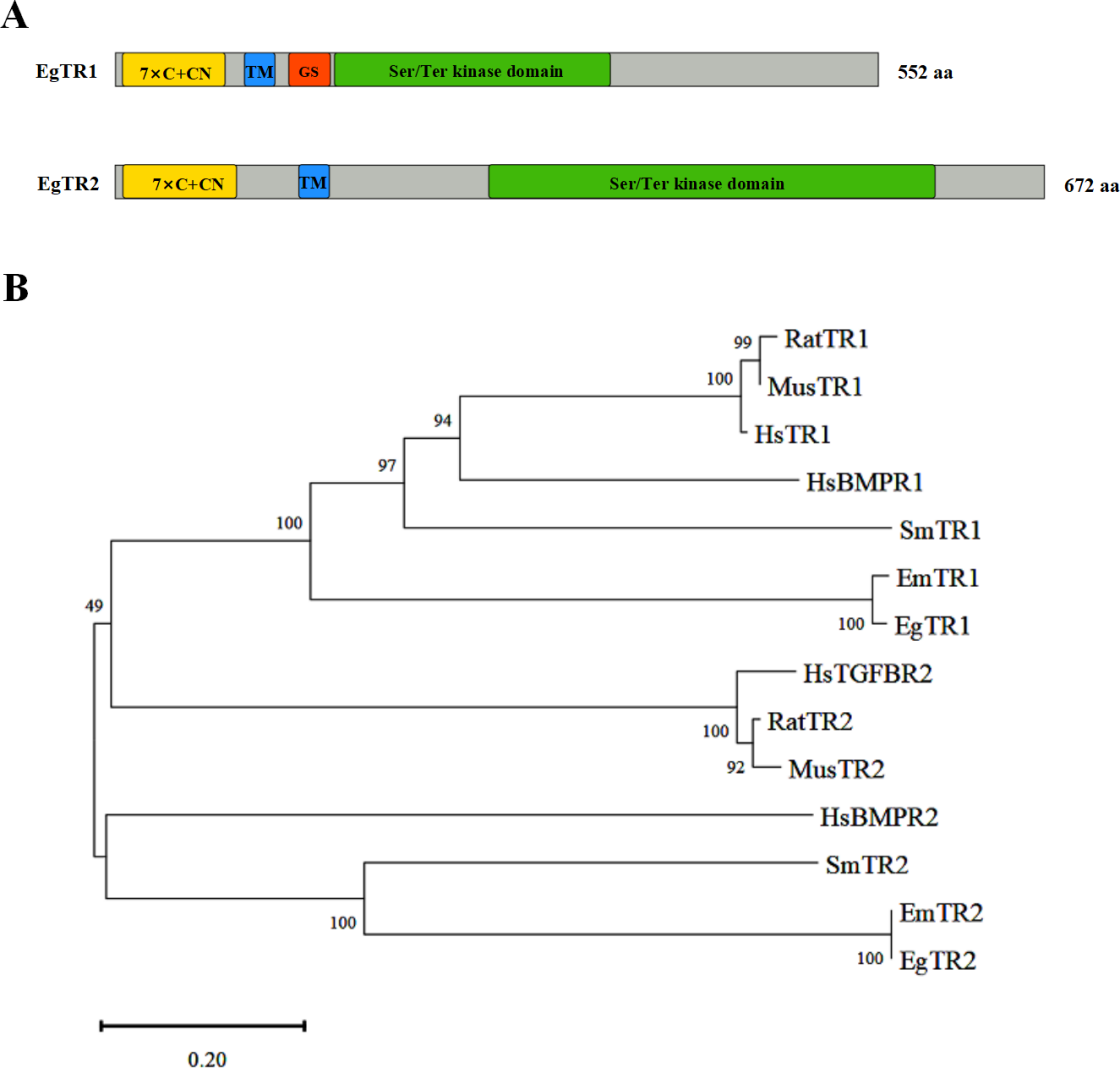
Structural and phylogenetic analysis of *E. granulosus s.s* TGFβ/BMP receptors. (**A**) Domain architectures of EgTR1 (type I) and EgTR2 (type II) receptors. Color-coded functional domains include cysteine-rich extracellular regions (yellow; 7 conserved cysteines with CC-CN motif), transmembrane domain (blue), GS box (red orange), and serine/threonine kinase domain (green). (**B**) Unrooted neighbor-joining phylogenetic tree constructed using MEGA11 (v11.0.13) with 1,000 bootstrap replicates. Full-length protein sequences were aligned via ClustalW, demonstrating evolutionary relationships between EgTR homologs and vertebrate TGFβ/BMP receptors. GenBank accession numbers: *E. granulosus s.s*., EgTR1, CAJ43247; *E. granulosus s.s*., EgTR2, PQ724305; *E. multilocularis*, EmTR1, CAH56492; *E. multilocularis*, EmTR2, CAQ76822; *S. mansoni*, SmTR1, AAC16404; *S. mansoni*, SmTR2, AAV67030; *M. musculus*, MusTR1, AAA40495; *M. musculus*, MusTR2, AAK98605; *R. norvegicus*, RatTR1, AAA83216; *R. norvegicus*, RatTR2, NP_112394; *H. sapiens*, HsTR1, ACZ58375; *H. sapiens*, HsTR2, NP_001020018; *H. sapiens*, HsBMPR1, BAA19765; *H. sapiens*, HsBMPR2, KAI4037675.

To identify TGFβ/BMP receptor homologs, specific primers were designed for amplifying sequences from *E. granulosus s.s*. Subsequently, two open reading frames (ORFs) cDNA fragments were amplified, and sequence analysis revealed 97.29%, 97.97% similarity to EmTR1 and EmTR2, respectively. The full-length ORF of EgTR1 (GeneBank accession number: CAJ43247) comprised 1,656 bp, encoding a 552 amino acid protein with a predicted molecular mass of 61.2 kDa (Fig. 1A). Computational algorithms predict that EgTR1 protein structure consists of a cysteine-rich receptor binding domain, a transmembrane domain, and a serine/threonine kinase domain with typical GS-box (motif SGSGSG) in the intracellular domain (Fig. S1). Similarly, the full-length ORF of EgTR2 (GeneBank accession number: PQ724305) comprised 2,016 bp, encoding a 672 amino acid protein with a predicted molecular mass of 74.2 kDa (Fig. 1A). The EgTR2 protein structure includes a receptor binding domain at the N-terminus, a transmembrane domain, and a serine/threonine kinase domain at the C-terminus (Fig. S2). The phylogenetic tree was constructed using the neighbor-joining method, incorporating TGFβ receptors and BMP receptors. Amino acid sequence alignments performed with DNAMAN software (Version 9.0) revealed high evolutionary conservation of EgTR1 and EgTR2. EgTR1 shared 96.93% identity with EmTR1, while EgTR2 exhibited 97.62% identity with EmTR2. (Fig. 1B).

### EgTR1 and EgTR2 were involved in dynamic transcriptional regulation during *E. granulosus s.s*. development

**Fig 2:**
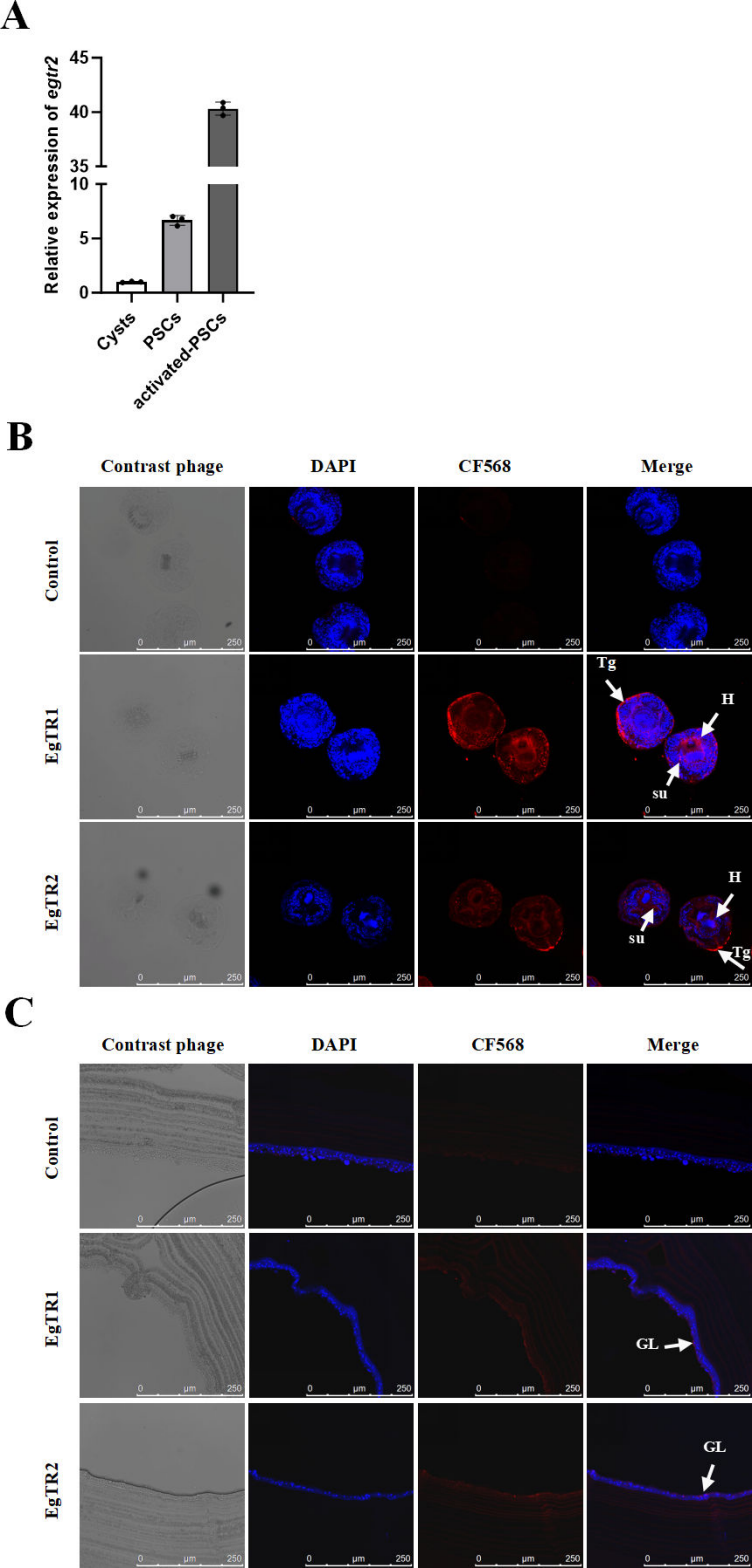
Subcellular localization of EgTR1 and EgTR2 in *E. granulosus* larval stages. (**A**) Quantitative PCR analysis was performed on total RNA isolated from cysts, protoscoleces (PSCs), and activated-PSCs. A bar graph comparing the relative expression levels (means ± SD) of *egtr2* normalized to the levels of *egelp*. The relative expression value was averaged from triple samples, and the experiment was repeated three times. (**B**) Immunofluorescence localization in PSCs, and (**C**) *in vitro*-cultivated cysts. Sections were incubated with affinity-purified EgTR1-immunized mice sera and EgTR2-immunized mice sera, respectively, and F(ab’)2 goat anti-mouse antibody conjugated to CF568. Controls were incubated with preimmunized mice sera. Nuclei were stained with DAPI (4′,6-diamidino-2-phenylindole dihydrochloride, blue). Cells positive (CF568, red) for EgTR1 or EgTR2 are indicated by white closed arrows. Tg, tegument; H, hooks; su, suckers; GL, germinal layer; LL, laminated layer.

The transcriptional expression levels of *egtr2* transcripts were assessed through quantitative reverse transcription polymerase chain reaction (qRT-PCR) across larval development stages. The levels of *egtr2* transcription varied among the three stages (activated PSCs, PSCs, and cysts), with the highest levels observed in activated PSCs and the lowest in cysts. The *egtr2* transcripts were 40-fold more abundant in activated PSCs than in cysts and 6.6-fold higher in activated PSCs compared to the PSCs (Fig. 2A). Consistent with previous reports on *egtr1* expression (18), these data highlight dynamic transcriptional regulation of TGFβ receptors during development. To further characterize the expression distribution of EgTR1 and EgTR2, an immunofluorescence analysis was conducted using mice anti-sera. The results showed that EgTR1 and EgTR2 were predominantly localized in the tegument tissue, sucker, and hooks in invaginated PSCs. Similarly, in *in vitro* cultured cysts, EgTR1 and EgTR2 were observed in the cytoplasm within the germinal layer. No fluorescence signals were observed in negative controls (Fig. 2B and C).

### Yeast two-hybrid analysis revealed functional domain localization of EgTR2-EgTR1 interactions and host-parasite ligand binding

**Fig 3:**
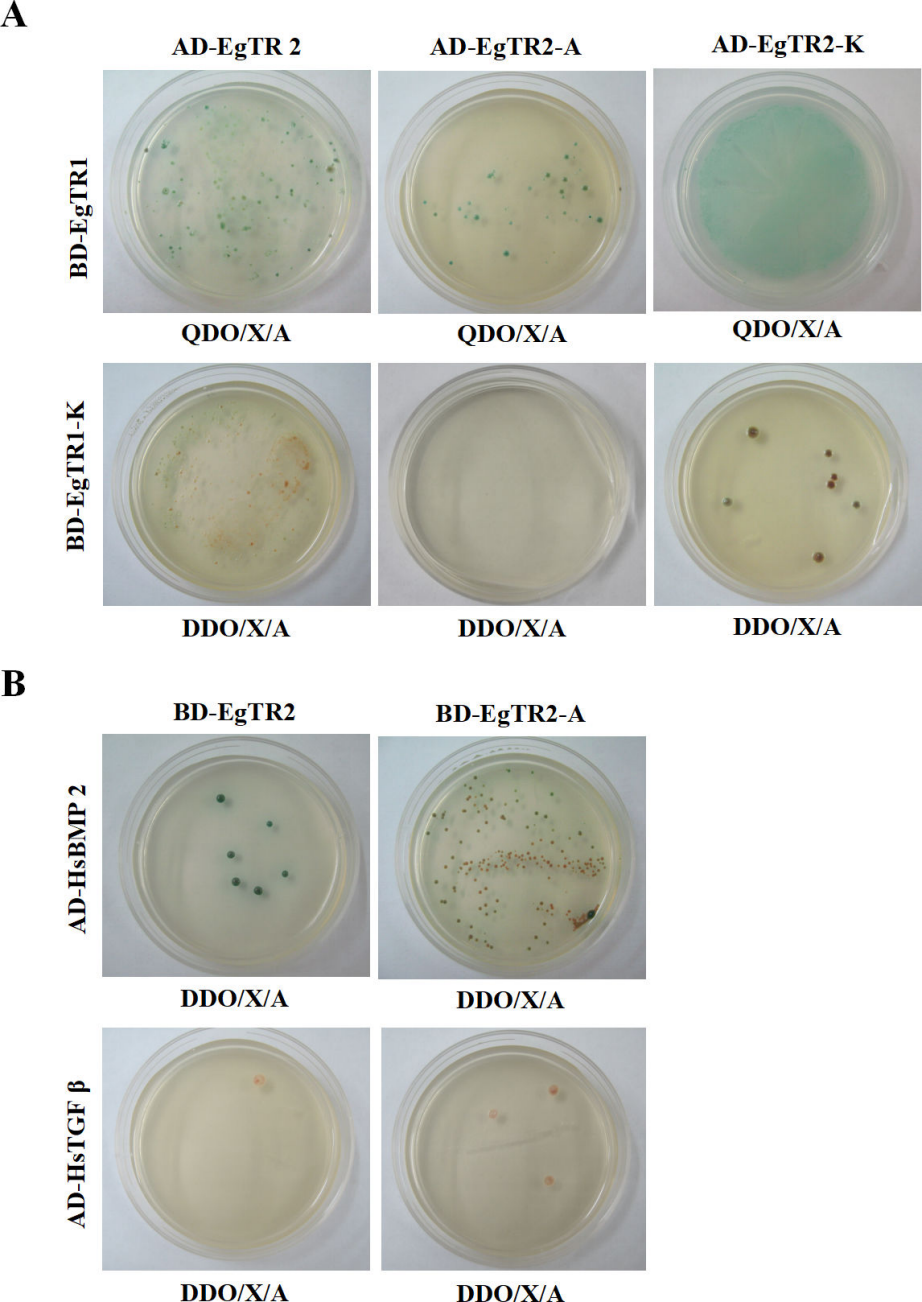
Yeast two-hybrid analysis of interactions between EgTR1, EgTR2, and signaling partners. (**A**) Interaction analysis between EgTR1 and EgTR2 proteins. (**B**) Interaction analysis between EgTR2 and human homologs (HsBMP2, HsTGFβ). Translational fusion constructs were generated by cloning EgTR1, EgTR2, HsTGF-β, and HsBMP2 into the Gal4 activation domain (pGADT7 vector) or Gal4 DNA binding domain (pGBKT7 vector) vectors. Yeast double transformants (strain Y2HGold) were cultured on selective media for 5 days at 30°C. High stringency conditions: SD/-Trp/-Leu/-Ade/-His/X-gal AbA (QDO/X-α-Gal/AbA); Low stringency conditions: SD/-Trp/-Leu/X-gal AbA (DDO/X-α-Gal/AbA).

The yeast two-hybrid system was utilized to investigate the interactions between EgTR1 and EgTR2, as well as human TGFβ/BMP ligands. Bait and prey vectors were co-transformed into the yeast strain Y2H Gold, and interactions were assessed on selective media. pGADT7-EgTR2 (AD-EgTR2) and pGBKT7-EgTR1 (BD-EgTR1) transformants exhibited strong interactions under high-stringency conditions (synthetically defined [SD]/-Trp/-Leu/-Ade/-His/X-α-gal/AbA), while AD-EgTR2 and BD-EgTR1-K (the intracellular kinase domain) transformants displayed weak interactions under low-stringency conditions (SD/-Trp/-Leu/X-α-gal/AbA). Additionally, to investigate the host-parasite interactions, human TGFβ and BMP2 were cloned as fusions with the Gal4 activation domain, while EgTR2 and the extracellular ligand-binding activation domain (EgTR2-A) were fused to the Gal4 binding domain and co-transformed into the yeast strain Y2H Gold. The results indicated that AD-HsBMP2 interacted with both EgTR2 and EgTR2-A, as evidenced by the transformants grown under low-stringency conditions (SD/-Trp/-Leu/X-α-gal/AbA) (Fig. 3 and Table 1; Fig. S3).

**TABLE 1:** Summary of EgTR2 interaction with EgTR1 and HsBMP2 analyzed by yeast two-hybrid assay^*a*^

Vector	Growth with interaction partner		
	AD-EgTR2	AD-EgTR2-A	AD-EgTR2-K
BD-EgTR1	+ +	+	+ +
BD-EgTR1-K	+	−−	+
	BD-EgTR2	BD-EgTR2-A	ND
AD-HsBMP2	+	+	ND
AD-HsTGF-β	−−	−−	ND

^*a*^ Summary of interaction network detected through yeast two-hybrid assay. Interaction status: (++), growth under high stringency; (+), growth under low stringency; (−−), no growth. ND, not determined.

### TGFβ/BMP pathway inhibitor LDN193189 inhibited the viability of *E. granulosus s.s*. *in vitro*

**Fig 4:**
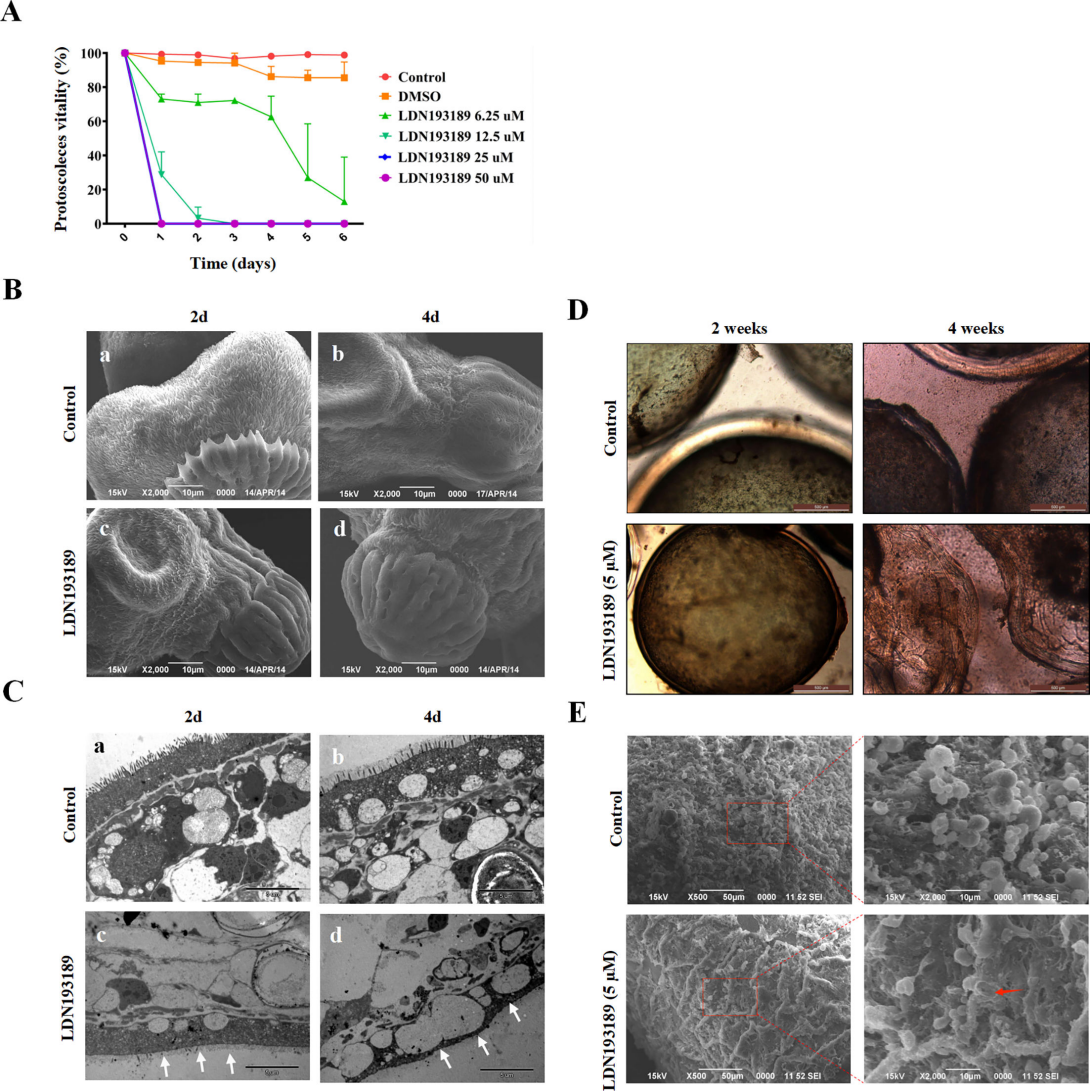
Effects of TGFβ/BMP receptor inhibitors on *E. granulosus s.s*. protoscoleces (PSCs) viability and hydatid cyst morphology. (**A**) Dose-dependent inhibition of PSCs viability. PSCs were treated with increasing concentrations of LDN193189 (6.25–50 µM) for 6 days. Cell viability was quantified using eosin Y exclusion assay (1%, wt/vol). Data represent mean ± SD of three independent experiments (*n* = 3). Controls: Untreated PSCs (Control) and 1% DMSO-treated PSCs (DMSO). (**B and C**) Ultrastructural alterations in PSCs. Morphological changes were analyzed using Scanning electron microscopy (SEM) (**B**) and transmission electron microscopy (TEM) (**C**) after incubation with either DMSO (control) or inhibitors 2, 4 days. (**D**) Cyst development inhibition. Cysts were incubated with 5 µM LDN193189 for 2 and 4 weeks, and the morphological changes of cysts were observed using light microscopy. (**E**) Ultrastructural changes of cysts detected by SEM after incubation with either DMSO (control) or inhibitors (LDN193189, 5 µM) 4 weeks. Red arrow indicates the ultrastructural changes.

Previous evidence highlights the pivotal role of TGFβ/BMP signaling in echinococcus anterior pole morphogenesis and protoscolex development (22). To further validate the role of TGFβ/BMP signaling in the growth and development of *E. granulosus s.s.,* PSCs and cysts were cultured *in vitro* with or without LDN193189. As illustrated in Fig. 4, PSCs exhibited striking dose-dependent susceptibility to LDN193189. Specifically, after 1 day of incubation, PSC viability was 95.28%, 73.13%, 28.76%, 0%, and 0% at LDN193189 concentrations of 0, 6.25, 12.5, 25, and 50 µM, respectively. By day 3, viability declined to 97.17%, 72.25%, 0%, 0%, and 0% at the same concentrations. On day 5, only 6.25 µM LDN193189 group retained partial viability to 26.97%, while all other treated groups showed complete lethality. In contrast, the untreated control group maintained >96% throughout the 5-day period (Fig. 4A). Morphological alterations in PSCs including tegumental alterations such as hooks loss and soma contraction observed via scanning electron microscope (SEM) (Fig. 4B). Transmission electron microscope (TEM) revealed ultrastructural changes such as microtriches shedding, cytoplasmic vacuolization, nuclear chromatin condensation, and the presence of residual lamellar bodies (Fig. 4C). For *in vitro* cultured cysts, LDN193189 treatment (5 µM for 4 weeks) induced structural collapse, including reduced turgor, germinal layer detachment from the laminated layer, and residual cellular debris (Fig. 4D). SEM confirmed micromorphological disruption, with the germinal layer fully detached from the acellular laminated layer. In contrast, DMSO-treated cysts retained intact architecture (Fig. 4E).

### LDN193189 pretreatment impairs *E. granulosus s.s*. cyst development *in vivo*

**Fig 5:**
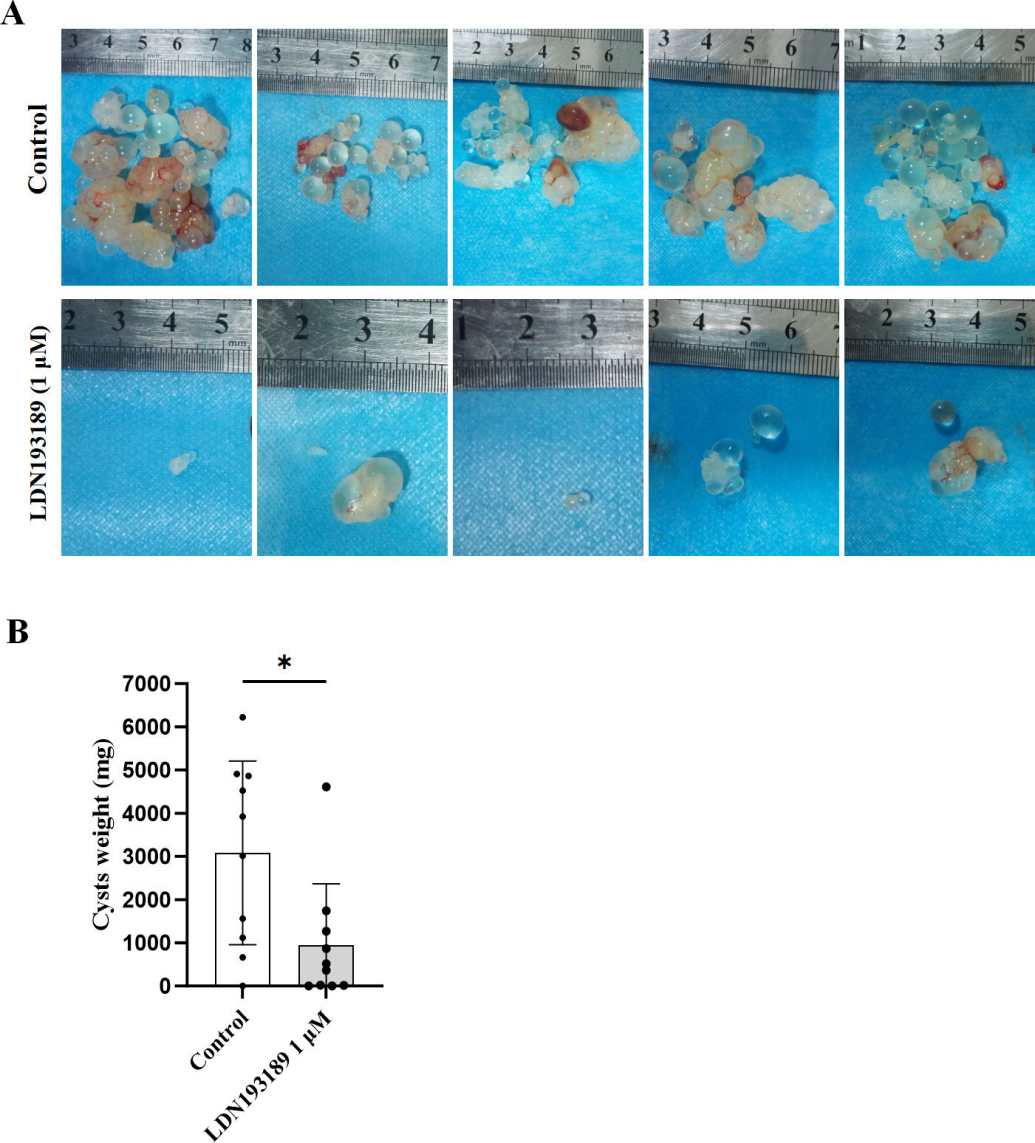
*In vivo* efficacy of LDN193189 against *E. granulosus s.s*. cyst development in BALB/c mice. (**A**) Representative hydatid cysts harvested 6 months post-intraperitoneal inoculation with PSCs. Pretreatment conditions: 30-day *in vitro* exposure to 1 µM LDN193189 (test) or DMSO (control). (**B**) Quantitative analysis of cyst biomass (wet weight). Bar graph indicating the distribution of cyst weights (milligrams) in the different treatment groups (*P* = 0.0165). Data are presented as mean ± SD. Statistical comparisons between control and LDN193189 were performed using Welch’s unequal variances *t*-test (*P ≤ 0.05).

To assess the impact of TGFβ/BMP pathway inhibitors on PSCs, *E. granulosus s.s*. PSCs were pretreated to 1 µM LDN193189 *in vitro* for 30 days, followed by a mouse bioassay to assess the efficacy. Following 6 months after intraperitoneal inoculation with the pretreated PSCs, the mean cyst significantly decreased from 3,078.85 ± 2,017.05 mg (DMSO control) to 940.39 ± 1,349.65 mg (LDN193189-pretreated group, *P =* 0.0165)*.* These findings demonstrate that pharmacological inhibition of the TGFβ/BMP pathway significantly impairs parasite survival and cyst formation *in vivo* (Fig. 5).

## DISCUSSION

The TGFβ/BMP signaling pathway, an evolutionarily conserved pathway, plays a critical role in modulating cellular proliferation, tissue morphogenesis, and organ development across species (23). Previous studies have elucidated the functional involvement of the TGFβ/Smad signaling cascade in diverse biological processes in schistosomes, including mating behavior, vitelline cell maturation, and egg embryogenesis (9, 24–26). Notably, in *Echinococcus multilocularis*, TGFβ/BMP signaling components are essential in anterior pole formation and PSC development (14, 15). While these pathways have been implicated as therapeutic targets for cancer and infectious diseases, the TGFβ receptors in *E. granulosus* s.s. remained uncharacterized.

In this study, we identified and functionally characterized two TGFβ receptors in *E. granulosus* s.s., designated as EgTR1 and EgTR2. Sequence alignment via BLASTp revealed that EgTR1 shares high homology with TGFβ receptor subgroup members in multiple species, particularly *Schistosoma mansoni* TGFβ receptors and *E. multilocularis* EmTR1. EgTR2, a novel type II receptor, exhibited close similarity to *E. multilocularis* EmTR2. Structural analysis demonstrated that both receptors possess a conserved extracellular domain featuring a Cys-box with seven flanking cysteine residues, coupled with an intracellular serine/threonine kinase domain. Notably, EgTR2 lacks the canonical GS-box characteristic of type I receptors, thus confirming its classification within the TGFβ/BMP type II receptor family.

Yeast two-hybrid assays demonstrated robust interactions between EgTR2 and both EgTR1 and HsBMP2. These findings align with prior reports of cross-species receptor-ligand interactions in *E. multilocularis*, where heterodimerization of EmTR1 with human bone morphogenetic protein type II receptor (BMPRII) activates BMP-regulated Smads (EmSmadB/EmSmadE) upon BMP2 stimulation (13, 14). Functional studies suggest that a single type II receptor can activate multiple type I receptors, enabling host-derived TGFβ/BMP ligands to initiate downstream Smad signaling (15). For instance, human TGF-β1 induces SmTR2-mediated activation of SmTR1 in schistosomes, promoting SmSmad2/SmSmad4 heterodimerization and subsequent regulation of gynecophoral canal protein (GCP) expression (9). In *E. granulosus* s.s., previous work identified the type I receptor EgTR1 and four Smad proteins (EgSmadA/C/D/E) (16–18, 27). Notably, EgSmadE undergoes nuclear translocation *in vitro* following exposure to human TGF-β1 or BMP2 (18). In this research, we observed elevated EgTR2 expression in activated PSCs compared to cysts or quiescent PSCs, which is consistent with the expression patterns of other TGFβ/Smad pathway components (18). Immunolocalization studies localized EgTR1 and EgTR2 to the tegument, sucker, and hooks of PSCs, and hydatid cyst germinal layer, suggesting their involvement in host-parasite interface dynamics, a phenomenon further supported by host-derived cytokine interactions (28–30).

Current CE management relies on surgical resection and benzimidazole (BZ) chemotherapy though BZ toxicity underscores the need for novel therapies. Kinase inhibitors have emerged as promising candidates (1, 31–35). We evaluated LDN193189, a BMP receptor kinase inhibitor, against *E. granulosus s.s.* PSCs. LDN193189 significantly reduced PSC viability and induced ultrastructural damage, including germinal layer detachment from the laminated layer. Additional inhibitors targeting specific pathways—WZB117 (GLUT1 inhibitor) and LDN212854 (BMPRI inhibitor)—demonstrated efficacy against *E. granulosus s.s.* and *E. multilocularis*, respectively (36, 37). These findings underscore inhibitors as potent anti-parasitic agents, offering novel strategies for CE treatment.

In conclusion, this study characterized two TGFβ/BMP signaling molecules of *E. granulosus s.s.* and examined the interaction of EgTR2 with EgTR1, and human BMP2, respectively. TGFβ/BMP receptor kinase would be a potential target for anti-parasitic drugs, such as LDN193189. Thus, further adjustments of treatment are to explore, such as optimal dosage and off-target effects require further investigation. In addition, new TGFβ/BMP signaling inhibitors offer starting points for the development of novel chemotherapeutic agents.

## MATERIALS AND METHODS

### Parasite material and *in vitro* cultivation

Protoscoleces (PSCs) were aseptically extracted from cysts of freshly slaughtered sheep infected with *E. granulosus s.s.* and cultured *in vitro*, as previously described (38). Activation of the PSCs using pepsin was conducted following the established method, which involved incubating the larvae in a low-pH solution (0.5 mg/mL pepsin in 0.85% [wt/vol] sodium chloride, pH 2.0, 37°C) for 30 min. The *in vitro* cultivation of cysts under axenic conditions was carried out using a previously established protocol (38).

### Nucleic acid isolation, cloning, and sequencing

Total RNA was isolated from *in vitro* cultured PSCs and cysts, as described in a previous study (16). The cDNA was synthesized using the RevertAid first-strand cDNA Synthesis Kit (Thermofisher Scientific, USA) according to the manufacturer’s instructions. Based on the conserved regions of EmTR1 (GeneBank accession number: AJ841786.1, TGFβ receptor I of *E. multilocularis*) and EmTR2 (GeneBank accession number: FM178547.1, TGFβ receptor II of *E. multiloculari*s), in conjunction with two homologous sequences in the supercontig database of *E. granulosus* (http://www.sanger.ac.uk/cgi-bin/blast/submitblast/Echinococcus), we identified two high-scoring segment pairs from *E. granulosus s.l.*. Specific primers used for EgTR1 and EgTR2 amplification were shown in Table S1. PCR products were cloned using the PCR cloning kit (TaKaRa, China) and then ligated to a pMD19-T Vector (TaKaRa, China). Positive clones were subsequently sequenced (Sangon Biotech Inc., China).

### Gene structure and phylogenetic analysis

To identify the gene structures of *egtr1* and *egtr2*, we analyzed the partial sequences available in the supercontig database of *E. granulosus s.l.* (http://www.sanger.ac.uk/cgi-bin/blast/submitblast/Echinococcus), using BLASTN with the *egtr1* and *egtr2* cDNA sequences. The phylogenetic analysis was constructed based on the predicted amino acid sequences of EgTR1, EgTR2, and other TGFβ/BMP receptors retrieved from GeneBank datebase (accessible via NCBI; https://www.ncbi.nlm.nih.gov) using the MEGA program (Version 11.0.13) as previously described (39). The transmembrane region of the conserved domain of EgTR2 was predicted using TMHMM (Version 2.0), and the conserved domain was identified using GenBank tools (40).

### Expression analysis in larval developmental stages

Quantitative reverse transcription polymerase chain reaction (qRT-PCR) was conducted to measure the gene transcriptional expression levels of *egtr1* and *egtr2* at various developmental stages. Total RNA extracted from *E. granulosus s.s.* PSCs and cysts were reverse transcribed, and the resulting cDNA was used as the templates for qRT-PCR amplification with SYBR Green PCR master mix (QIAGEN, Germany). Specific primers used for *egtr1*, *egtr2*, and *egelp* (used as an internal control) amplification are shown in Table S1. qRT-PCRs were performed in triplicate (final volume of 20 µL), on a CFX96 PCR system (Bio-Rad Inc.). The experimental protocol followed the manufacturer’s instructions, including an initial denaturation at 95°C for 2 min, 40 cycles of denaturation at 95°C for 15 s, annealing at 60°C for 30 s, melt curve analysis. Relative gene expression was calculated using the 2^−∆∆CT^ method with *egelp* as the normalization control.

### Preparation of mouse anti-sera and immunolocalization analysis

The open reading frame (ORF) of EgTR1 (1,656 bp, 552 amino acids) and EgTR2 (2,016 bp, 672 amino acids) were cloned into pET-28α (+), respectively. Recombinant His-tagged proteins expressed in *Escherichia coli* BL21 were affinity-purified and used for mice immunization, following a previously established immunization strategy (21). Pre-immune mice sera served as negative controls. Eight weeks after the final immunization, the anti-sera were collected, and the serum titer was determined by enzyme-linked immunosorbent assay (ELISA), demonstrating high titers (>1:256,000 for EgTR1 and >1:512,000 for EgTR2) (Fig. S4).

Immunolocalization of EgTR1 and EgTR2 was performed on frozen sections of fresh PSCs and cysts following established protocols (18). The sections were fixed for 15 min, immersed in blocking buffer (10% BSA) for 30 min, washed with phosphate-buffered saline (PBS), and incubated overnight at 4°C with EgTR1 and EgTR2 anti-sera, respectively (1:200 dilution). Subsequently, the sections were washed with PBS and incubated with the fluorochrome-conjugated secondary antibody (Alexa Fluor 568, Thermo Fisher Scientific Inc., USA) for 2 h at room temperature in darkness. Nuclei were stained with 4,6-diamidino-diamidino-2-phenylindole (DAPI, Cell Signaling Technology, USA) for 5 min. Imaging was performed using a confocal laser scanning fluorescence microscope (Leica TCS SP8, Germany). The primary anti-sera were replaced by pre-immunized mice sera in the negative control.

### Yeast two-hybrid analysis of EgTR1-EgTR2 interaction and ligand specificity

Yeast two-hybrid assays were used to assess the binding interaction between EgTR1, EgTR2, and hsBMP2. The full-length EgTR2, the extracellular ligand-binding activation domain (EgTR2-A, residues 1–128), and the intracellular kinase domain (EgTR2-K, residues 268–600) were cloned into the vectors pGADT7 (AD-EgTR2, AD-EgTR2-A, and AD-EgTR2-K), and pGBKT7 (BD-EgTR2, BD-EgTR2-A and BD-EgTR2-K), respectively. Similarly, HsTGFβ and HsBMP2 were cloned into the vector pGADT7 (AD-HsTGFβ and AD-HsBMP2). In parallel, full-length EgTR1 and its intracellular domain (EgTR1-K) were cloned into pGBKT7 (BD-EgTR1 and BD-EgTR1-K), respectively. The interaction assays were carried out by co-transformation of bait and prey plasmids into the yeast strain Y2HGold, following a previously described protocol (18).

### *In vitro* effects of LDN193189 on *E. granulosus s.s.* viability

*E. granulosus s.s.* PSCs and cysts were obtained as previously described (18). Cysts with diameters of 2–4 mm were selected for *in vitro* experimental studies. Viable PSCs were seeded in 96-well cell culture plates, while cysts were seeded in 6-well plates and cultured in RPMI 1640 medium containing 10% fetal bovine serum, 100 U/mL penicillin, and 100 µg/mL streptomycin. The culture was maintained in a 5% CO_2_ atmosphere at 37°C.

After 3 days of normal culture, PSCs with viability exceeding 95% were divided into different groups: (i) DMSO group, treated with 0.2% (vol/vol) DMSO and (ii) inhibitor groups, treated with gradient concentrations of the BMP pathway inhibitor LDN193189 (50 µM, 25 µM, 12.5 µM, 6.25 µM, Selleck Chemicals, Shanghai, China). The PSCs were incubated at 37°C in 5% CO_2_, and the medium was replaced every 3–4 days. Viability of PSCs was assessed by 1% eosin staining combined with morphological analysis. For ultrastructural analysis, PSCs and cysts treated with LDN193189 were collected and examined using scanning electron microscopy (SEM) (LEO1430VP, ZEISS, Germany) and transmission electron microscopy (TEM) (JEM-1230, Hitachi, Japan) as previously described (22).

### Assessment of *E. granulosus s.s.* PSCs' viability *in vivo*

To evaluate the effectiveness of LDN193189-treated PSCs, female BALB/c mice (6 weeks old, specific pathogen-free) were intraperitoneally injected 1,000 LDN193189-treated PSCs (pre-treated *in vitro* with 1 µM LDN193189). The control group received PSCs treated with PBS containing 0.2% DMSO. After 6 months of infection, the mice were euthanized, and the weight of parasite tissue was measured.

### Statistical analysis

Results were expressed as mean ± standard deviation (SD) and analyzed using GraphPad Prism 10.0 (GraphPad Software, San Diego, CA). Continuous variables were analyzed by Student’s *t*-test (normally distributed data) or Kruskal-Wallis’s test (non-parametric data), with *P <* 0.05 considered statistically significant.

## SUPPLEMENTAL MATERIAL

Supplemental material spectrum.01318-25-s0001.pdf Fig S1 to S4; Table S1.

ASM does not own the copyrights to Supplemental Material that may be linked to, or accessed through, an article. The authors have granted ASM a non-exclusive, world-wide license to publish the Supplemental Material files. Please contact the corresponding author directly for reuse.

## References

[1] Wen H, Vuitton L, Tuxun T, Li J, Vuitton DA, Zhang W, McManus DP. 2019. Echinococcosis: advances in the 21st Century. Clin Microbiol Rev 32:e00075-18. doi:10.1128/CMR.00075-18 30760475 PMC6431127

[2] Zhang W, Zhang Z, Wu W, Shi B, Li J, Zhou X, Wen H, McManus DP. 2015. Epidemiology and control of echinococcosis in central Asia, with particular reference to the People’s Republic of China. Acta Trop 141:235–243. doi:10.1016/j.actatropica.2014.03.014 24686096

[3] Casulli A. 2021. New global targets for NTDs in the WHO roadmap 2021-2030. PLoS Negl Trop Dis 15:e0009373. doi:10.1371/journal.pntd.0009373 33983940 PMC8118239

[4] Woolsey ID, Miller AL. 2021. Echinococcus granulosus sensu lato and Echinococcus multilocularis: a review. Res Vet Sci 135:517–522. doi:10.1016/j.rvsc.2020.11.010 33246571

[5] Massagué J, Sheppard D. 2023. TGF-β signaling in health and disease. Cell 186:4007–4037. doi:10.1016/j.cell.2023.07.036 37714133 PMC10772989

[6] Wrana JL, Attisano L, Wieser R, Ventura F, Massagué J. 1994. Mechanism of activation of the TGF-β receptor. Nature 370:341–347. doi:10.1038/370341a0 8047140

[7] Derynck R, Zhang YE. 2003. Smad-dependent and Smad-independent pathways in TGF-β family signalling. Nature 425:577–584. doi:10.1038/nature02006 14534577

[8] Kang JS, Liu C, Derynck R. 2009. New regulatory mechanisms of TGF-beta receptor function. Trends Cell Biol 19:385–394. doi:10.1016/j.tcb.2009.05.008 19648010

[9] Osman A, Niles EG, Verjovski-Almeida S, LoVerde PT. 2006. Schistosoma mansoni TGF-β receptor II: role in host ligand-induced regulation of a schistosome target gene. PLoS Pathog 2:e54. doi:10.1371/journal.ppat.0020054 16789838 PMC1479047

[10] Freitas TC, Jung E, Pearce EJ. 2007. TGF-β signaling controls embryo development in the parasitic flatworm Schistosoma mansoni. PLoS Pathog 3:e52. doi:10.1371/journal.ppat.0030052 17411340 PMC1847691

[11] Zavala-Góngora R, Kroner A, Wittek B, Knaus P, Brehm K. 2003. Identification and characterisation of two distinct Smad proteins from the fox-tapeworm Echinococcus multilocularis. Int J Parasitol 33:1665–1677. doi:10.1016/s0020-7519(03)00208-x 14636682

[12] Zavala-Góngora R, Derrer B, Gelmedin V, Knaus P, Brehm K. 2008. Molecular characterisation of a second structurally unusual AR-Smad without an MH1 domain and a Smad4 orthologue from Echinococcus multilocularis. Int J Parasitol 38:161–176. doi:10.1016/j.ijpara.2007.07.008 17845804

[13] Epping K, Brehm K. 2011. Echinococcus multilocularis: molecular characterization of EmSmadE, a novel BR-Smad involved in TGF-β and BMP signaling. Exp Parasitol 129:85–94. doi:10.1016/j.exppara.2011.07.013 21802416

[14] Zavala-Góngora R, Kroner A, Bernthaler P, Knaus P, Brehm K. 2006. A member of the transforming growth factor-beta receptor family from Echinococcus multilocularis is activated by human bone morphogenetic protein 2. Mol Biochem Parasitol 146:265–271. doi:10.1016/j.molbiopara.2005.12.011 16434111

[15] Kaethner M, Epping K, Bernthaler P, Rudolf K, Thomann I, Leitschuh N, Bergmann M, Spiliotis M, Koziol U, Brehm K. 2023. Transforming growth factor-β signalling regulates protoscolex formation in the Echinococcus multilocularis metacestode. Front Cell Infect Microbiol 13:1153117. doi:10.3389/fcimb.2023.1153117 37033489 PMC10073696

[16] Li J, Zhang CS, Lv GD, Wang JH, Wei XF, Lin RY, Yan GQ. 2011. Cloning and preliminary characterization of a SmadA gene from Echinococcus granulosus. Acta Vet Zootech Sin 42:1756–1762.

[17] Li J, Zhang CS, Lv GD, Wang JH, Wei XF, Li CW, Feng JL, Lin RY, Yan GQ. 2012. Construction of DNA-AD and DNA-BD vectors with EgSmadC and MH2 domain for yeast two-hybrid system. Chin J Vet Sci 32:58–62. doi:10.16303/j.cnki.1005-4545.2012.01.016

[18] Zhang C, Wang L, Wang H, Pu H, Yang L, Li J, Wang J, Lü G, Lu X, Zhang W, Vuitton DA, Wen H, Lin R. 2014. Identification and characterization of functional Smad8 and Smad4 homologues from Echinococcus granulosus. Parasitol Res 113:3745–3757. doi:10.1007/s00436-014-4040-4 25039015

[19] Li J, Li L, Zhang CS, Ye JW, Bi XJ, Lv GD, Lin RY. 2017. Construction and identification of DNA-AD and DNA-BD vectors for EgSmadE in a yeast two-hybridsys-tem. J Pathog Biol 12:535–539. doi:10.13350/j.cjpb.170612

[20] Yang L, Wang LM, Zhang CS, Li L, Wang JH, Lv GD, Wang H, Wen H, Lin RY. 2013. Construction of a prokaryotic expression vector containing the intracellular domain of the transforming growth factor beta type I receptor of Echinococcus granulosus and purification of the fusion protein. J Pathog Biol 8:1089–1092. doi:10.13350/j.cjpb.2013.12.013

[21] Li L, Li J, Zhang CS, Lv GD, Bi XJ, Lin RY. 2016. Construction of the fusion protein pET28a-EgTβRII-E and production of antiserum against Echinococcus granulosus. J Pathog Biol 11:719–722. doi:10.13350/j.cjpb.160810

[22] Ceballos L, Elissondo C, Sánchez Bruni S, Denegri G, Lanusse C, Alvarez L. 2011. Comparative performances of flubendazole and albendazole in cystic echinococcosis: ex vivo activity, plasma/cyst disposition, and efficacy in infected mice. Antimicrob Agents Chemother 55:5861–5867. doi:10.1128/AAC.05105-11 21930885 PMC3232756

[23] Shi Y, Massagué J. 2003. Mechanisms of TGF-β signaling from cell membrane to the nucleus. Cell 113:685–700. doi:10.1016/s0092-8674(03)00432-x 12809600

[24] Carlo JM, Osman A, Niles EG, Wu W, Fantappie MR, Oliveira FMB, LoVerde PT. 2007. Identification and characterization of an R-Smad ortholog (SmSmad1B) from Schistosoma mansoni. FEBS J 274:4075–4093. doi:10.1111/j.1742-4658.2007.05930.x 17635586

[25] Freitas TC, Jung E, Pearce EJ. 2009. A bone morphogenetic protein homologue in the parasitic flatworm, Schistosoma mansoni. Int J Parasitol 39:281–287. doi:10.1016/j.ijpara.2008.08.001 18765241 PMC2852128

[26] Buro C, Oliveira KC, Lu Z, Leutner S, Beckmann S, Dissous C, Cailliau K, Verjovski-Almeida S, Grevelding CG. 2013. Transcriptome analyses of inhibitor-treated schistosome females provide evidence for cooperating Src-kinase and TGFβ receptor pathways controlling mitosis and eggshell formation. PLoS Pathog 9:e1003448. doi:10.1371/journal.ppat.1003448 23785292 PMC3681755

[27] Yang L, Wang LM, Zhang CS, Li L, Wang JH, Lv GD, Wang H, Lin RY, Wen H. 2013. Full-length and intracellular domain constructs of transforming growth factor -β typeI receptor from Echinococcus granulosus for use in a yeast two-hybrid system and testing of the bait plasmid for autoactivation. J Pathog Biol 8:988–992. doi:10.13350/j.cjpb.2013.11.022

[28] Hemer S, Konrad C, Spiliotis M, Koziol U, Schaack D, Förster S, Gelmedin V, Stadelmann B, Dandekar T, Hemphill A, Brehm K. 2014. Host insulin stimulates Echinococcus multilocularis insulin signalling pathways and larval development. BMC Biol 12:5. doi:10.1186/1741-7007-12-5 24468049 PMC3923246

[29] Förster S, Koziol U, Schäfer T, Duvoisin R, Cailliau K, Vanderstraete M, Dissous C, Brehm K. 2019. The role of fibroblast growth factor signalling in Echinococcus multilocularis development and host-parasite interaction. PLoS Negl Trop Dis 13:e0006959. doi:10.1371/journal.pntd.0006959 30849083 PMC6426264

[30] Feng C, Cheng Z, Xu Z, Tian Y, Tian H, Liu F, Luo D, Wang Y. 2022. EmCyclinD-EmCDK4/6 complex is involved in the host EGF-mediated proliferation of Echinococcus multilocularis germinative cells via the EGFR-ERK pathway. Front Microbiol 13:968872. doi:10.3389/fmicb.2022.968872 36033888 PMC9410764

[31] Chiodini PL. 2023. Medical management of cystic echinococcosis. Curr Opin Infect Dis 36:303–307. doi:10.1097/QCO.0000000000000947 37593991

[32] Koike A, Becker F, Sennhenn P, Kim J, Zhang J, Hannus S, Brehm K. 2022. Targeting Echinococcus multilocularis PIM kinase for improving anti-parasitic chemotherapy. PLoS Negl Trop Dis 16:e0010483. doi:10.1371/journal.pntd.0010483 36190997 PMC9560627

[33] Ayala-Aguilera CC, Valero T, Lorente-Macías Á, Baillache DJ, Croke S, Unciti-Broceta A. 2022. Small molecule kinase inhibitor drugs (1995-2021): medical indication, pharmacology, and synthesis. J Med Chem 65:1047–1131. doi:10.1021/acs.jmedchem.1c00963 34624192

[34] Yang WB, Luo F, Zhang W, Sun CS, Tan C, Zhou A, Hu W. 2023. Inhibition of signal peptidase complex expression affects the development and survival of Schistosoma japonicum. Front Cell Infect Microbiol 13:1136056. doi:10.3389/fcimb.2023.1136056 36936776 PMC10020623

[35] Schiedel M, McArdle DJB, Padalino G, Chan AKN, Forde-Thomas J, McDonough M, Whiteland H, Beckmann M, Cookson R, Hoffmann KF, Conway SJ. 2023. Small molecule ligands of the BET-like bromodomain, SmBRD3, affect Schistosoma mansoni survival, oviposition, and development. J Med Chem 66:15801–15822. doi:10.1021/acs.jmedchem.3c01321 38048437 PMC10726355

[36] Amahong K, Yan M, Li J, Yang N, Liu H, Bi X, Vuitton DA, Lin R, Lü G. 2021. EgGLUT1 is crucial for the viability of Echinococcus granulosus sensu stricto metacestode: a new therapeutic target? Front Cell Infect Microbiol 11:747739. doi:10.3389/fcimb.2021.747739 34858873 PMC8632494

[37] Li J, Li DW, Li L, Yang ST, Hou XL, Wang H, Zhang CS. 2021. The effect of the bone morphogenetic protein inhibitor LDN-212854on Echinococcus multilocularis protoscoleces in vitro. J Pathog Biol 16:1280–1284. doi:10.13350/j.cjpb.211109

[38] Zhang WB, Jones MK, Li J, McManus DP. 2005. Echinococcus granulosus: pre-culture of protoscoleces in vitro significantly increases development and viability of secondary hydatid cysts in mice. Exp Parasitol 110:88–90. doi:10.1016/j.exppara.2005.02.003 15804383

[39] Zhang C, Li J, Aji T, Li L, Bi X, Yang N, Li Z, Wang H, Mao R, Lü G, Shao Y, Vuitton DA, Wen H, Lin R. 2019. Identification of functional MKK3/6 and MEK1/2 homologs from Echinococcus granulosus and investigation of protoscolecidal activity of mitogen-activated protein kinase signaling pathway inhibitors in vitro and in vivo. Antimicrob Agents Chemother 63. doi:10.1128/AAC.01043-18 PMC6325220 30348669

[40] Gulyaeva AA, Sigorskih AI, Ocheredko ES, Samborskiy DV, Gorbalenya AE. 2020. LAMPA, LArge Multidomain Protein Annotator, and its application to RNA virus polyproteins. Bioinformatics 36:2731–2739. doi:10.1093/bioinformatics/btaa065 32003788 PMC7203729

