# Supplementary material for: Functional characterization of TGFβ/BMP receptors in Echinococcus granulosus sensu stricto: implications for parasite survival and therapeutic targeting
Source: Microbiol Spectr. 2025 Nov 26;14(1):e01318-25. doi: 10.1128/spectrum.01318-25 (PMC12772319; doi:10.1128/spectrum.01318-25)
Supplement: Supplemental material — Fig S1 to S4; Table S1. [file spectrum.01318-25-s0001.pdf]

Figure S1. Characterization of *E. granulosus s. s.*TGF-β type I receptor.

(A) Electrophoresis of the amplified PCR products of EgTR1 gene. EgTR1 gene fragment of 1,656 bp was cloned from PSCs. M, DL2000; 1, EgTR1 gene; Two exons interrupted by one intron constitute the EgTR1 gene. (B) Multiple-sequence alignments of EgTR1 from *E. granulosus s.s.*; *Echinococcus multilocularis* (GenBank: AJ841786.1); *Schistosoma japonicum* (GenBank: KAH8870216.1); *Mus musculus* (GenBank: L15436.1); *Rattus norregicus* (GenBank: L26110.1); *Homo sapiens* (GenBank: AK302234.1).

Figure S2. Characterization of *E. granulosus s. s.* TGF-β type II receptor.

(A) Electrophoresis of the amplified PCR products of EgTR2 gene. EgTR2 gene fragment of 2,016 bp was cloned from PSCs. Marker (M, DL2000) and EgTR2 gene (lane 1); Seven exons interrupted by six introns constitute the EgTR2 gene. (B) Multiple-sequence alignments of EgTR2 from *E. granulosus s.s.* (GenBank: XNZ84554.1); *Echinococcus multilocularis* (GenBank: CAQ76822.1); *Schistosoma japonicum* (GenBank: ACZ05060.1); *Mus musculus* (GenBank: BAA06840.1); *Rattus norregicus* (GenBank: AAA42237.1); *Homo sapiens* (GenBank: KAI2528689.1). Extracellular regions (Green), transmembrane domain (Red), Intracellular regions (Purple).

Figure S3. Yeast two-hybrid analysis of interactions between EgTR1 and EgTR2

(A) Growth of cotransformed Y2HGold yeast cells on selective DDO/X/AbA (EgTR2

and EgTR1, EgTR2-A and EgTR1, EgTR2-K and EgTR1, respectively). (B) No-growth of cotransformed Y2HGold yeast cells on selective QDO/X/AbA (EgTR2 and EgTR1-K, EgTR2-K and EgTR1-K, respectively). (C) No-growth of cotransformed Y2HGold yeast cells on selective QDO/X/AbA (HsBMP2 and EgTR2, HsBMP2 and EgTR2, respectively). DDO/X/AbA (synthetically defined /-Trp/-Leu/X-gal AbA); QDO/X/AbA (synthetically defined /-Trp/-Leu/-Ade/-His/X-gal AbA);

Figure S4. ELISA analysis of anti-sera of mouse immunized by EgTR1 and EgTR2 recombinant proteins

(A) Sera of five immunized mice were analyzed for ELISA titer of EgTR1, and EgTR2 (B). Sera of pre-immune mice were used as negative control (n=5).

Table S1. List of primer sequences

Fig. S1

A

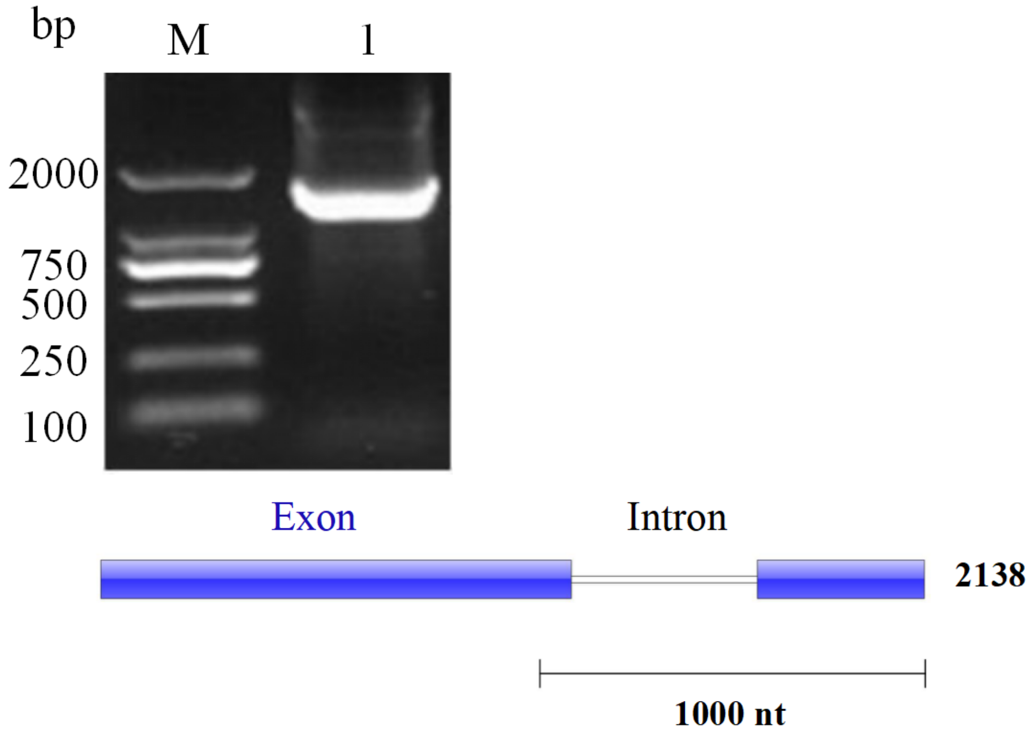
bp
M
1
2000
750
500
250
100
Exon
Intron
2138
1000 nt

B

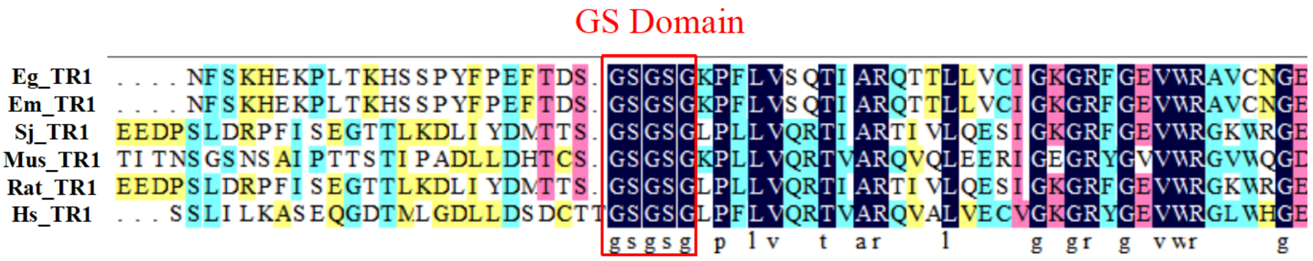
GS Domain
Eg_TR1 ....NFSKHEKPLTKHSSPYFPEFTDS.GSGSGKPFLVSQTIARQTTLLVCIGKGRFGEVWRAVCNGE
Em_TR1 ....NFSKHEKPLTKHSSPYFPEFTDS.GSGSGKPFLVSQTIARQTTLLVCIGKGRFGEVWRAVCNGE
Sj_TR1 EEDPSLDRPFISEGTTLKDLIYDMTTS.GSGSGLPLLVQRTIARTIVLQESIGKGRFGEVWRGKWRGE
Mus_TR1 TITNSGSNSAIPTTSTIPADLLDHTCS.GSGSGKPLLVQRTVARQVQLEERIGEGRYGVVWRGVWQGD
Rat_TR1 EEDPSLDRPFISEGTTLKDLIYDMTTS.GSGSGLPLLVQRTIARTIVLQESIGKGRFGEVWRGKWRGE
Hs_TR1 ...SSLILKASEQGDTMLGDLLDSDCTTGSGSGLPFLVQRTVARQVALVECVGKGRYGEVWRGLWHGE
gsgsg p lv t ar l g gr g vwr g

Fig. S2

A

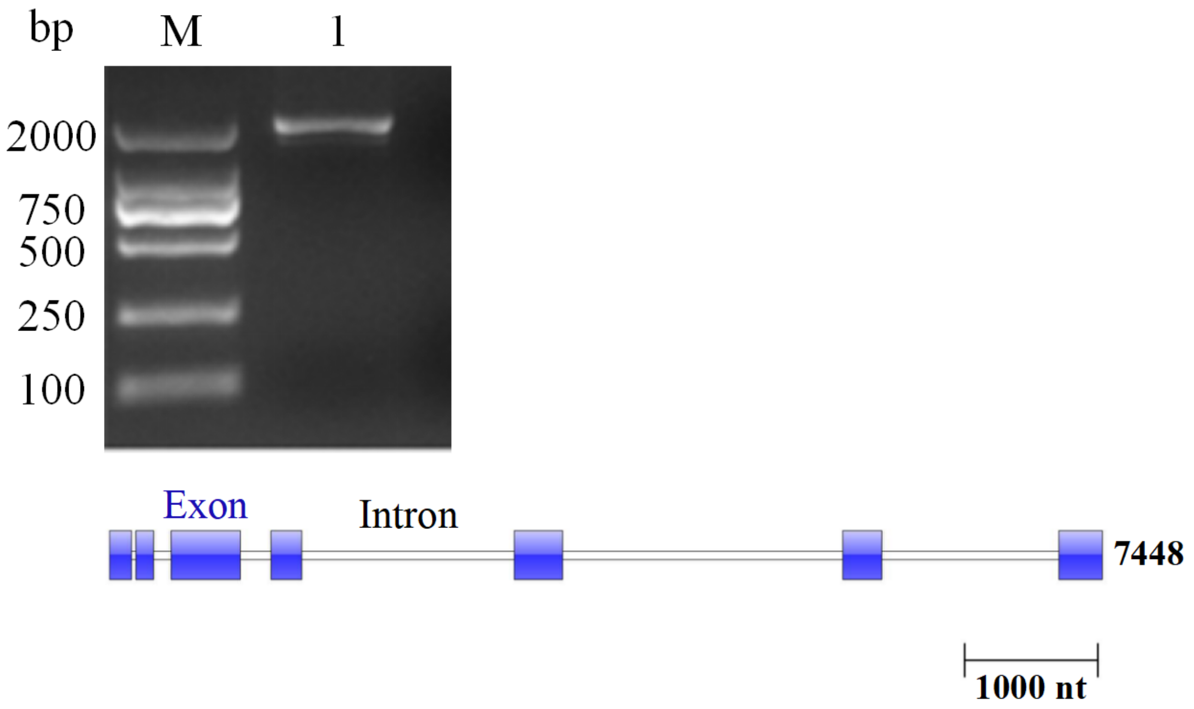
bp
M
1
2000
750
500
250
100
Exon
Intron
7448
1000 nt

B

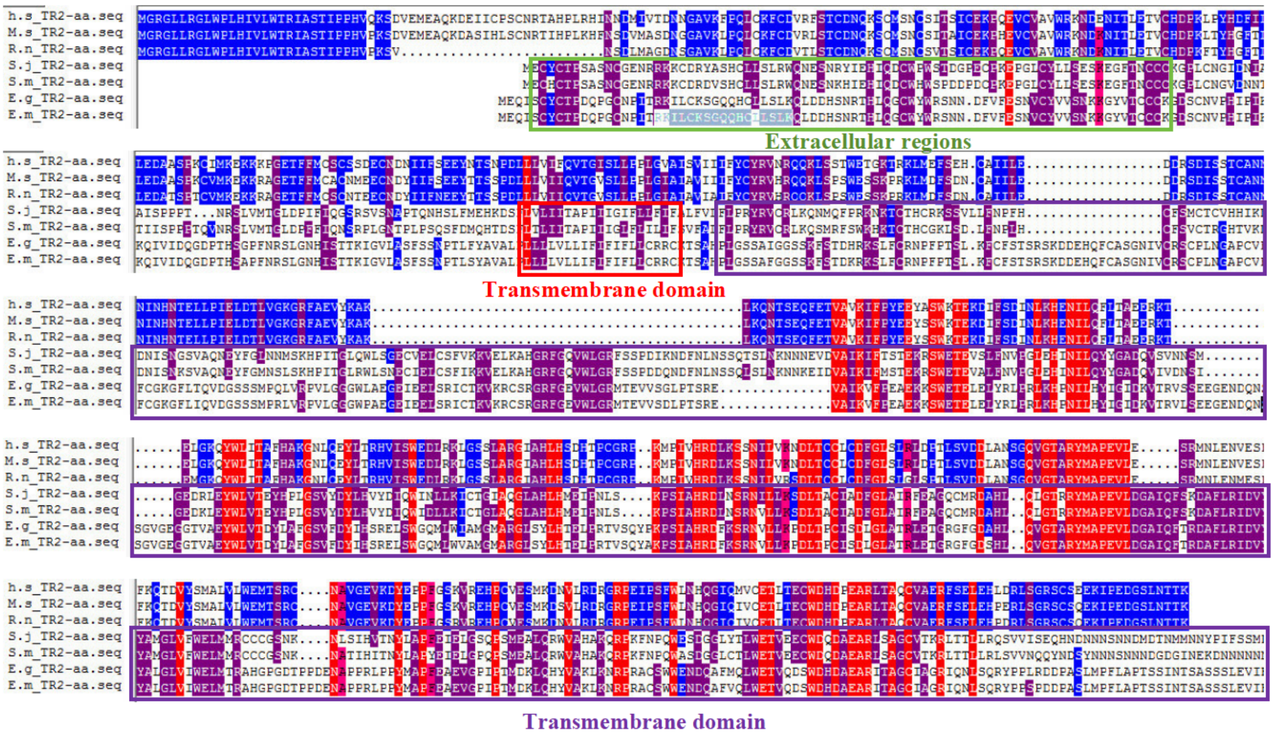
h.s_TR2-aa.seq
M.s_TR2-aa.seq
R.n_TR2-aa.seq
S.j_TR2-aa.seq
S.m_TR2-aa.seq
E.g_TR2-aa.seq
E.m_TR2-aa.seq
Extracellular regions
Transmembrane domain
Transmembrane domain

Fig. S3

A

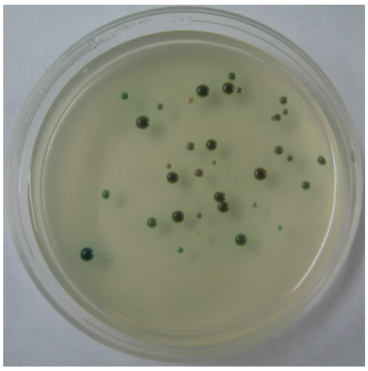

AD-EgTR2
BD-EgTR1
DDO/X/AbA

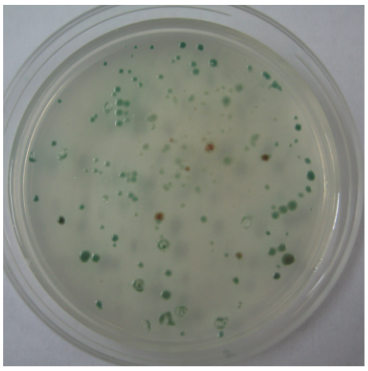

AD-EgTR2-A
BD-EgTR1
DDO/X/AbA

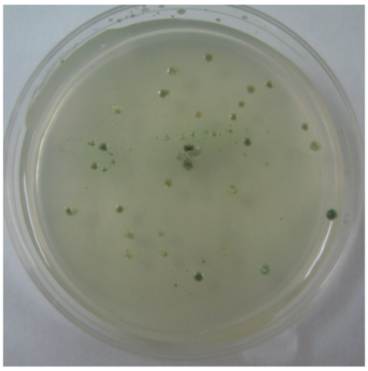

AD-EgTR2-K
BD-EgTR1
DDO/X/AbA

B

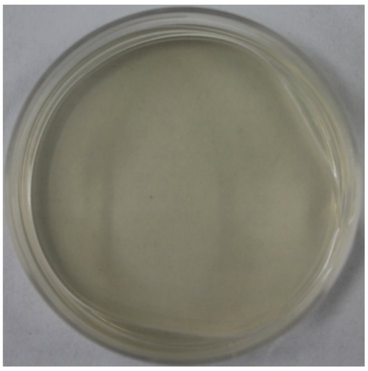

AD-EgTR2
BD-EgTR1-K
QDO/X/AbA

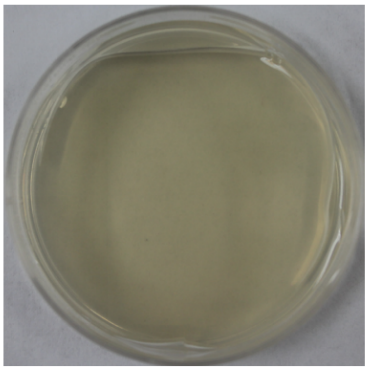

AD-EgTR2-K
BD-EgTR1-K
QDO/X/AbA

C

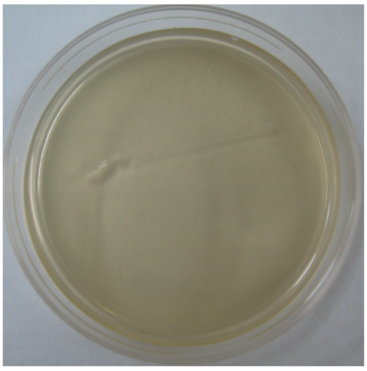

AD-HsBMP2
BD-EgTR2
QDO/X/AbA

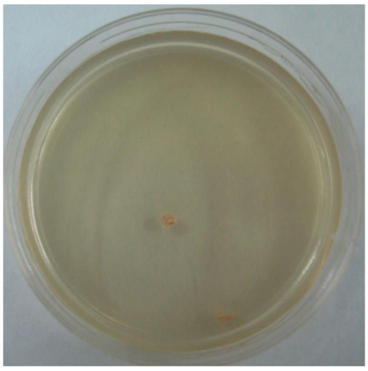

AD-HsBMP2
BD-EgTR2-A
QDO/X/AbA

Fig. S4

A

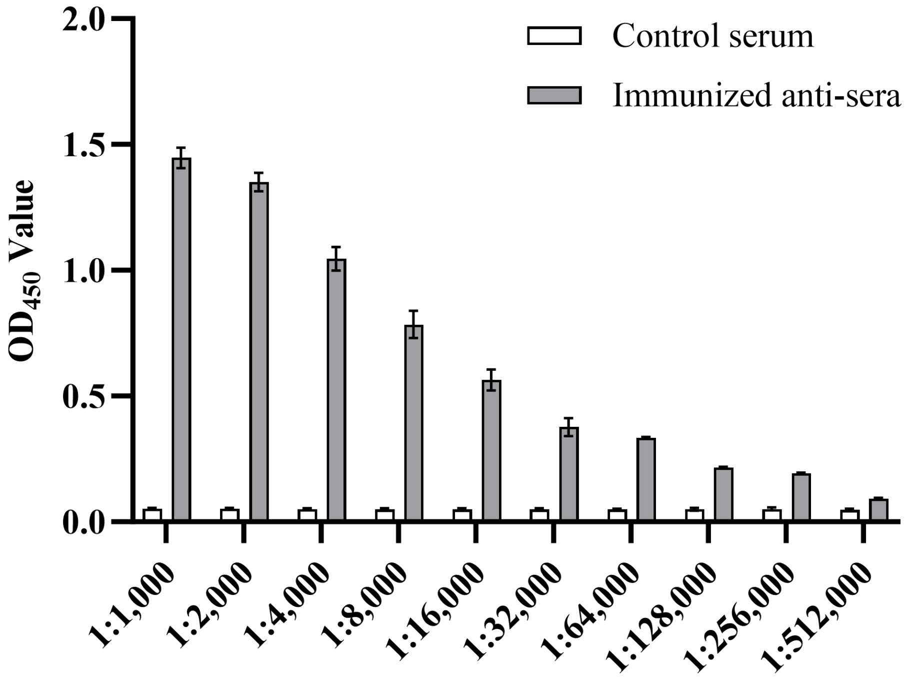

Control serum
Immunized anti-sera
OD450 Value
2.0
1.5
1.0
0.5
0.0
1:1,000
1:2,000
1:4,000
1:8,000
1:16,000
1:32,000
1:64,000
1:128,000
1:256,000
1:512,000

B

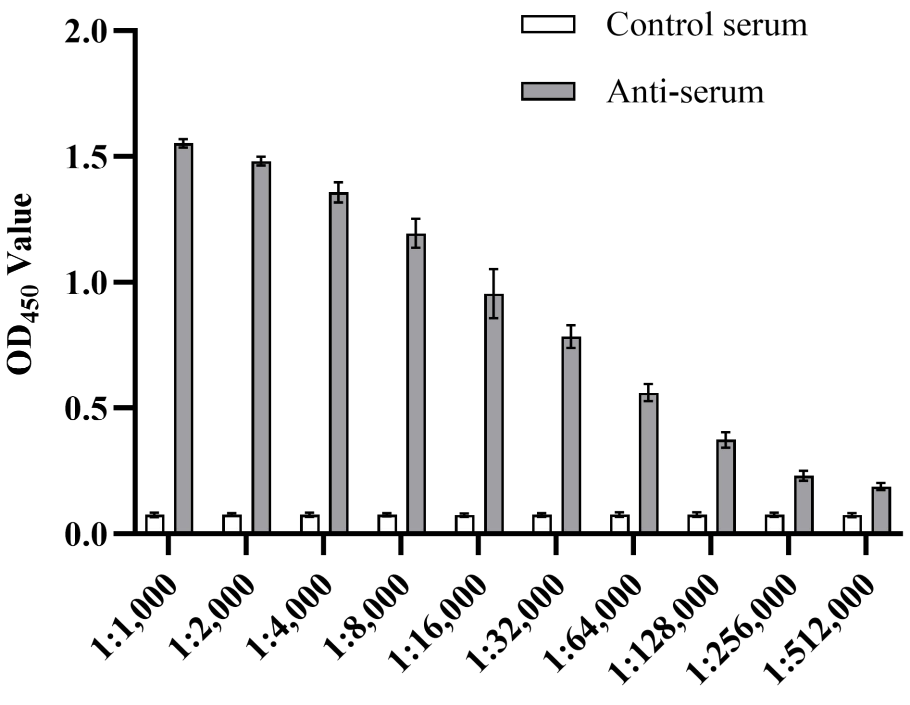

Control serum
Anti-serum
OD450 Value
2.0
1.5
1.0
0.5
0.0
1:1,000
1:2,000
1:4,000
1:8,000
1:16,000
1:32,000
1:64,000
1:128,000
1:256,000
1:512,000

Table S1

Primer	Sequence5’→3’(restriction sites under	Enzyme sites
EgTR1-F1	ATTCCCGGGGATGAAAAATCAT	
EgTR1-R1	AAAACTGCAGGGCTCACAGTCG	
EgTR1-K-F1	GTCGCATATGAAAGGGAACTTC	
EgTR1-K-R1	GTCGTCGACGGCTCACAGTCGC	
EgTR2-F1	TTGTGATTGTTTCTGGCCCTG	---
EgTR2-R1	TGGAACGAACTGCAAGGAGA	---
EgTR2-F(2)	CGGGATCCCGTGTAGTTATTTAT	Bam HI
EgTR2-R(2)	CAAGCTTGACCGCTGCTACACCT	Hind 2I
EgTR2out-F	CGCGGATCCGCGATGGAACAAA	Bam HI
EgTR2out-R	CCGCTCGAGCGGGAAGAGGGTT	Xho I
AD-TR2-A(F)	CGCGGATCCGCGATGGAACAAA	BamHI
AD-TR2-A(R)	CCGCTCGAGCGGGAAGAGGGTT	XhoI
AD-TR2-K(F)	CGCGGATCCGCGTCGAGGATAT	BamHI
AD-TR2-K(R)	CCGCTCGAGCGGATACCTTTGAC	XhoI
AD-TR2 -(F)	CGCGGATCCGCGATGGAACAAA	BamHI
AD-TR2 -(R)	CCGCTCGAGCGGATACCTTTGAC	XhoI
BD-TR2-A(F)	CGCGGATCCGCGATGGAACAAA	Nde I
BD-TR2-A(R)	GCACTGCAGTGCGAAGAGGGTT	PstI
BD-TR2-K(F)	CGCGGATCCGCGTCGAGGATAT	NdeI
BD-TR2-K(R)	GCACTGCAGTGCATACCTTTGAC	PstI
BD-TR2- (F)	CGCGGATCCGCGATGGAACAAA	NdeI
BD-TR2 -(R)	GCACTGCAGTGCATACCTTTGAC	PstI
AD-BMP2-(F)	CGCGGATCCGCGATGGTGGCCG	BamHI
AD-BMP2-(R)	CCGCTCGAGCGGCTAGCGACAC	XhoI
AD-TGF-(F)	CGCGGATCCGCGATGCACGTGC	BamHI
AD-TGF-(F)	CCGCTCGAGCGGCTAGTGGCAG	XhoI
